# An Engineered Yeast Expressing an Artificial Heavy Metal-Binding Protein Enhances the Phytoremediation of Alum Mine Soils

**DOI:** 10.3390/microorganisms13030612

**Published:** 2025-03-07

**Authors:** Wenming Wang, Liling Xie, Lin Zhao, Qilin Yu

**Affiliations:** 1 School of Environmental Science and Engineering, Tianjin University, Tianjin 300072, China; wangwenming1980@163.com (W.W.); zhaolin@tju.edu.cn (L.Z.); 2 National Key Laboratory of Intelligent Tracking and Forecasting for Infectious Diseases, College of Life Sciences, Nankai University, Tianjin 300071, China; 2120231478@mail.nankai.edu.cn

**Keywords:** heavy metal-binding protein, alum mine soil, artificial yeast, cadmium capture, soil quality

## Abstract

Alum mining leads to significant heavy metal and acid pollution within soils. Phytoremediation is a common strategy used to treat alum mine soils, but its efficiency is frequently compromised by the alum-mining-induced impairment of plant growth. To improve the strength of plants against mine pollution, this study constructed the artificial yeast strain ScHB (heavy metal-binding protein-containing *Saccharomyces cerevisiae*) expressing the de novo designed protein HBGFP (heavy metal-binding green fluorescence protein) and investigated its effect on the phytoremediation of alum mine soils with soil physiochemical assays and heavy metal quantification. This protein was composed of an N-terminal signal peptide, an HB (heavy metal-binding) domain, and a GFP (green fluorescence protein) domain, as well as a C-terminal glycolphosphatidylinositol-anchoring fragment. The exposure of the HBGFP on the ScHB surface increased the growth rate of the yeast cells and enhanced cadmium capture from the cadmium-containing medium. After culturing *Medicago sativa* in the alum mine soils for 30 days, ScHB remarkably increased the plants’ average height from 17.5 cm to 27.9 cm and their biomass from 3.03 g/plant to 4.35 g/plant, as well as increasing the accumulation of antioxidant agents in the plants. Moreover, the ScHB cells strongly improved the soil quality, with an increase in the soil pH values from 5.47 to 6.21 to 6.9, and increased the levels of soil organic matter, total nitrogen, available phosphorus, and living bacteria. Furthermore, ScHB efficiently improved the plants’ abilities to remove soil heavy metals, decreasing the levels of cadmium, lead, chromium, and copper by 90%, 86%, 97%, and 88%, respectively. This study developed a genetic engineering method to improve the efficiency of phytoremediation against pollution from alum mining.

## 1. Introduction

Mining is a critical process within industry development that provides essential materials for metal purification, mineral production, coal production, etc. [1,2,3]. However, this process is frequently followed by the release of heavy metals, organic pollutants, and other hazardous components, leading to the contamination of both soils and groundwater [4,5]. For example, alum mining may bring with it serious soil pollution in the form of different heavy metals and acidic minerals [6,7]. This pollution further inhibits plant growth, resulting in a disruption to mountain vegetation layers, soil erosion, and the dispersion of these pollutants [8,9,10]. Until now, the remediation of soils polluted in situ by the alum mining process has remained a great challenge.

Phytoremediation is no doubt a sustainable and environmentally friendly way to recover the soil quality in mining areas [11,12]. Remediation plants can transport the heavy metals from polluted soils to the plant’s body, reducing the heavy metal content in the soil [13,14]. These plants may also secrete organic nutrients around their roots, facilitating the accumulation of functional microbes within the rhizosphere [15,16,17]. The microbes further adsorb inorganic and organic pollutants, fix nitrogen, and solubilize mineral nutrients (e.g., phosphorus, potassium, and iron) [18,19]. A series of plants, including *Medicago sativa* (clover), *Solanum nigrum* (nightshade), and *Lactuca serriola* (wild lettuce), have been used for the phytoremediation of mining soils [20,21,22]. However, the efficiency of these plants remains to be further improved, especially when such plants are used to remedy mining soils containing toxic acids and heavy metals.

Genetic engineering of microbes is a promising strategy for the promotion of microbial activity [23,24]. A series of engineered microbes, e.g., Escherichia *coli*, *Pseudomonas putida*, and *Saccharomyces cerevisiae*, have been constructed to capture different kinds of pollutants [25,26,27]. For instance, *E. coli* cells were transformed using cadmium-capturing genes to enhance their cadmium tolerance and removal abilities [28]. This implies that the application of artificial microbes may improve phytoremediation during the treatment of mining soils. However, until now, no studies have reported on the application of engineered microbes to improve the phytoremediation of mining soils.

Therefore, this study aimed to construct an engineered yeast strain expressing a heavy metal-capturing protein and to explore its effect on the phytoremediation of alum mining soils with *M. sativa*. The heavy metal-binding protein contained a N-terminal signal peptide, an HB (heavy metal-binding) domain, a green fluorescence protein (GFP) domain, and a C-terminal glycolphosphatidylinositol-anchoring fragment. The capacity of the artificial yeast to tolerate and capture heavy metals was evaluated using a liquid cadmium-supplied medium. The effects of this yeast on plant growth, soil quality, and heavy metal removal during phytoremediation were further evaluated in the alum mining soils. This study sheds light on the application of genetic engineering in the phytoremediation of mining soils.

## 2. Materials and Methods

### 2.1. Materials

The initial *S. cerevisiae* strain Sc0, i.e., InvSc1, was purchased from Invitrogen, Carlsbad, CA, USA. Calcofluor White (CFW) was purchased from Sigma, St. Louis, MO, USA. CdCl_2_ was purchased from Aladdin, Shanghai, China. The organic matter (OM), total nitrogen (TN), and available phosphorus (AvP) assay kits were purchased from Jiahang, Beijing, China. The GSH and ascorbic acid assay kits were purchased from Jiancheng, Nanjing, China.

### 2.2. Construction of the Artificial Yeast Cells

The artificial yeast strain ScHB was constructed from the initial yeast strain Sc0. Firstly, the artificial protein HB (Table S1) was designed by fusing the N-terminal signal peptide (SP), the heavy metal-binding domain HB, the fluorescence protein GFP, and the C-terminal GPI fragment. The *HBGFP* gene of the artificial protein HBGFP was then designed using the optimal codons of *S. cerevisiae*. The DNA fragment of SHBGG was de novo synthesized by QYAOBIO, Shanghai, China, and then cloned into the *S. cerevisiae* expression plasmid pESCptef-URA [29], obtaining the plasmid pESCptef-HBGFP-URA. This plasmid was transformed into the initial *Saccharomyces cerevisiae* strain Sc0 using the lithium acetate-polyethylene glycol transformation method. The artificial yeast strain ScHB was screened on the plates of SC-Ura (yeast nitrogen base, 0.67 g; glucose, 2 g; amino acid mixture without uracil, 0.2 g; agar, 2 g; and distilled water, 100 mL). The artificial yeast cells were cultured in a liquid YPD medium (peptone, 2 g; glucose, 2 g; yeast extract, 1 g; distilled water, 100 mL) or a medium containing cadmium ion (5 mg/L) for further investigation.

### 2.3. Confocal Microscopy

To observe the expression and distribution of HBGFP in the yeast cells, both the Sc0 cells and ScHB cells were cultured for 24 h at 30 °C in the liquid YPD medium with shaking at 120 rpm. The cells were then harvested following centrifugation at 12,000 rpm for 2 min, washed twice with PBS (pH = 7.2), and re-suspended in PBS. The cells were stained with CFW (final concentration at 10 mg/L) for 5 min and observed via confocal microcopy (FV1000, Olympus, Tokyo, Japan).

### 2.4. SEM Observation and EDS Mapping

The yeast cells were cultured in a liquid YPD medium containing 5 mg/L cadmium ion (i.e., cadmium chloride at 8.1 mg/L) at 30 °C and shaken at 120 rpm for 24 h. The cells were then harvested following centrifugation, washed twice with distilled water, and fixed with 4% formaldehyde for 24 h. The fixed cells were dehydrated using ethanol and dried with a frozen vacuum drier. The dried cells were further observed via SEM (TESCAN MIRA LMSM, Brono, Czech Republic), and the element distribution in the yeast cells were analyzed using an EDS mapping model of the SEM images.

### 2.5. Plant Culturing

To investigate the effect of the yeast cells on plant growth, *M. sativa* seeds were added into the nutrient soil in seedling beds, followed by pre-culturing for 30 days. The obtained young plants, with heights of 10 cm ± 1 cm, were then transplanted into the alum mine soils. Four experimental groups were set: the control group, with no plant culturing and no addition of yeast cells; the Ms group, with plant culturing but no addition of the yeast cells; the Ms + Sc0 group, with plant culturing and the addition of Sc0 cells (1 × 10^8^ yeast cells/kg soil); and the Ms + ScHB group, with plant culturing and the addition of ScHB cells (1 × 10^8^ yeast cells/kg soil). After 30 days of further culturing with normal watering and sunlight at room temperature (25–30 °C), the plants were sampled, photos were taken, and their heights and weights (i.e., biomass) were measured.

### 2.6. Biochemical Analysis of the Plants

The chlorophyll contents of the *M. sativa* leaves were detected after 30 days of culturing. A chlorophyll analyzer (Laiyin, Weifang, China) was used to measure the chlorophyll levels in the plant leaves, with 100 leaves for each group measured. The leaf SPAD values of each leaf were recorded to indicate the chlorophyll levels.

To detect the reduced GSH and ascorbic acid levels in the leaves, 1 g of the sampled plant leaves was suspended in 5 mL of PBS buffer (pH = 7.2). The mixtures were added into glass homogenizers, and the leaves were damaged 50 times. The obtained homogenates were centrifuged at 12,000 rpm for 5 min, obtaining their corresponding supernatants. The contents of reduced GSH and ascorbic acid in the supernatants were detected using GSH and ascorbic acid assay kits, respectively.

### 2.7. Analysis of Soil Properties

The rhizosphere alum mine soils of the plants were sampled after 30 days of culturing. The control soil was sampled from the control group with no plant culturing and no addition of yeast cells. The pH values of the soil samples were measured via dispersion of 10 g of the soils into 50 mL of distilled water, followed by measurement of the supernatant pH value with a pH detector (Mettler Toledo, Zurich, Swissland). To analyze the other soil physiochemical properties, the sampled rhizosphere soils were dried using a freeze vacuum drier (Scientz-10N, Scientz, Ningbo, China) for 24 h. The organic matter, TN, and AvP contents in the sampled soils were then detected using OM, TN, and AvP assay kits, respectively.

### 2.8. CFU Assays

To measure the number of total and cadmium-tolerant bacteria, 1 g of the sampled soils in each group was thoroughly dispersed into 100 mL of distilled water in 250 mL sterilized flasks containing 20 g of glass beads. After 5 min of shaking in swing beds with 200 rpm at 20 °C, the suspensions were then diluted with a 10-fold gradient using distilled water. The diluted suspensions were added onto the LB medium (tryptone, 1 g; yeast extract, 0.5 g; sodium chloride, 1 g; agar, 2 g; distilled water, 100 mL) and the cadmium-containing LB medium (tryptone, 1 g; yeast extract, 0.5 g; sodium chloride, 1 g; agar, 2 g; cadmium chloride, 0.817 mg; distilled water, 100 mL), respectively. After culturing the plates in an incubator at 30 °C for 3 days, the CFUs were counted, obtaining the number of total and cadmium-tolerant bacteria, respectively.

### 2.9. Heavy Metal Quantification

The heavy metal contents of the soil samples were measured using the inductively coupled plasma (ICP) method. Briefly, 1 g of the soil samples was added into 10 mL of 30% HNO_3_ solution. After 1 h of organic matter digestion, the reaction liquids were centrifuged at 12,000 rpm for 2 min. The supernatants were diluted with deionized water, and the cadmium, lead, chromium, and copper contents were detected using an ICP analyzer (Optima 8300, PerkinElmer, Waltham, MA, USA).

### 2.10. Statistical Analysis

Each experiment was repeated three times. The results are shown as means ± standard deviations. Differences between two groups were evaluated using Student’s *t*-test (*p* < 0.05) via the SPSS software (version 22.0, IBM, Armonk, NY, USA).

## 3. Results

### 3.1. Construction of the Artificial Heavy Metal-Binding Yeast ScHB

**Figure 1:**
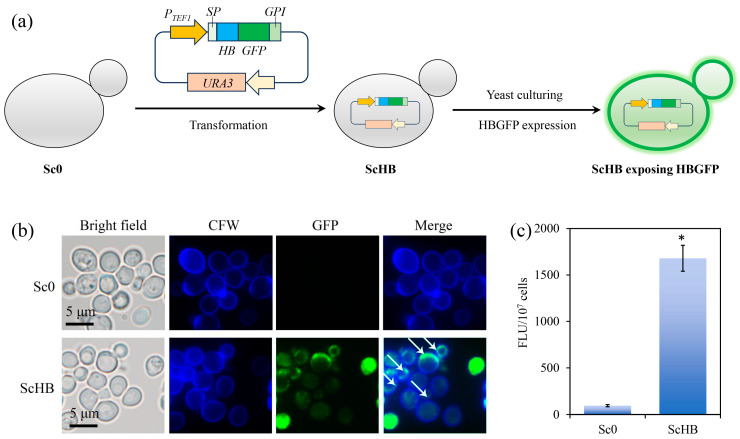
Construction of the artificial heavy metal-binding yeast ScHB. (**a**) A scheme illustrating the construction of ScHB. (**b**) Confocal observation of the control Sc0 cells and the ScHB cells. The white arrows indicate the exposure of HBGFP on the yeast cells. (**c**) Fluorescence intensity of the yeast cells. The asterisk (*) indicates significant difference between the groups (*p* < 0.05).

To promote heavy metal capture with remediation plants, the artificial heavy metal-binding yeast ScHB (heavy metal-binding protein-containing *Saccharomyces cerevisiae*) was constructed by designing an artificial protein HBGFP. This protein contains the N-terminal signal peptide (SP) of MFα1 [30], the heavy metal-binding domain HB [31], the fluorescence protein GFP, and the C-terminal glycolphosphatidylinositol-anchoring fragment (GPI) of AGα1 [32]. The presence of SP and GPI allows for the exposure of HB on the yeast cell surface [33,34]. The gene sequence of the artificial protein HBGFP was designed, synthesized, and cloned into the overexpression plasmid pESCptef-URA, obtaining the final plasmid pESCptef-HBGFP-URA. This plasmid was transformed into the initial *Saccharomyces cerevisiae* strain InvSc1 (i.e., Sc0), generating the artificial yeast strain ScHB for further yeast culturing and HBGFP expression (Figure 1a). As revealed via confocal microscopy, the control Sc0 cells had no obvious GFP fluorescence signal, while the artificial ScHB cells displayed strong GFP fluorescence (Figure 1b,c). Moreover, most of the HBGFP signals of the ScHB cells were co-localized with the fluorescence of Calcofluor White (CFW, indicating cell wall) (Figure 1b), indicating the main distribution of HBGFP on the cell surface. These results revealed that the ScHB cells strongly expressed the artificial protein and efficiently displayed this protein on their cell surface.

### 3.2. The Artificial ScHB Cells Exhibit Strong Cadmium-Capturing Capacity

**Figure 2:**
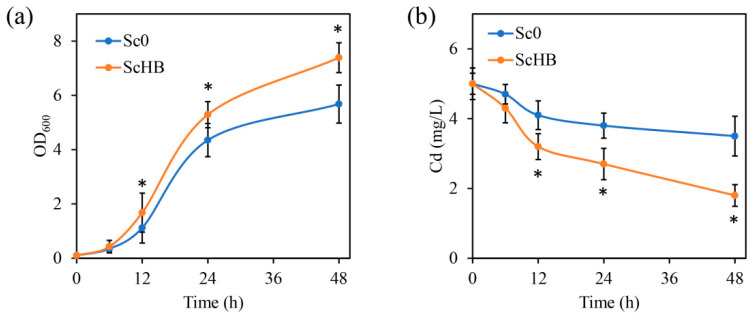
Growth curves (**a**) and cadmium-capturing capacity (**b**) of Sc0 and ScHB. The asterisks (*) indicate significant differences between the two strains (*p* < 0.05).

The surface exposure of the heavy metal-capturing protein HBGFP may lead to high tolerance to heavy metals and strong capture of these toxic ions. To confirm this, the yeast cells were cultured in YPD (yeast extract–peptone–dextrose) medium containing 5 mg/L of cadmium ion. With the increase in culturing time, both the Sc0 and ScHB cells grew rapidly for 6 to 24 h, and their growth tended to stabilize after 24 h. As compared with the Sc0 cells, the artificial ScHB cells had higher growth biomass at the same time points. For instance, after 24 h of growth, the OD_600_ value of the ScHB cells reached 5.3, while that of the Sc0 cells only reached 4.4 (Figure 2a). Furthermore, the ScHB cells captured higher levels of cadmium from the culturing medium than the Sc0 cells. After 24 h of incubation, ScHB reduced the cadmium contents in the liquid medium from 5 mg/L to 2.7 mg/L, while Sc0 only reduced the cadmium contents to 3.8 mg/L. More strikingly, after 48 h of treatment, ScHB exhibited a much higher cadmium-capturing percent than Sc0 (i.e., 64% versus 30%, Figure 2b). These results indicated that ScHB had a higher capacity to tolerate and capture heavy metal cadmium than Sc0.

**Figure 3:**
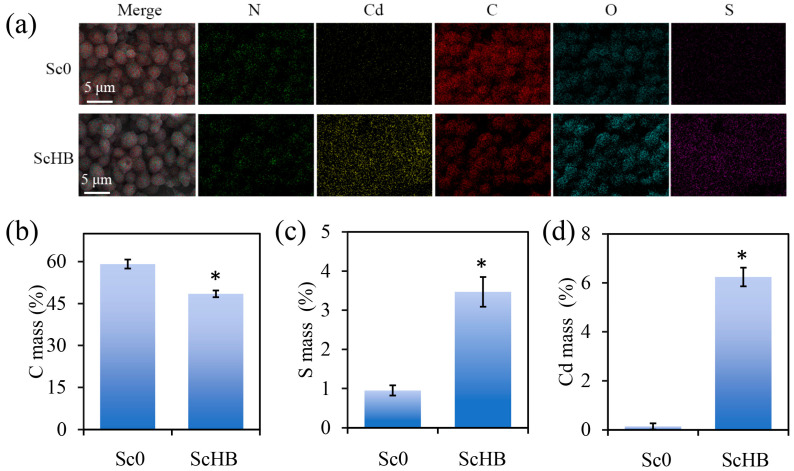
EDS mapping analysis of element distribution in the yeast cells. The yeast cells were cultured in YPD medium containing 5 mg/L of Cd^2+^ for 24 h, followed by centrifugation, drying, and EDS mapping analysis. (**a**) The EDS mapping images of the yeast cells. (**b**) The C mass percent. (**c**) The S mass percent. (**d**) The Cd mass percent. The asterisks (*) indicate significant differences between the two strains (*p* < 0.05).

An energy-dispersive X-ray spectrum (EDS) mapping analysis was performed to further confirm cadmium capture by the yeast cells. After 24 h of treatment, the Sc0 and ScHB cells were harvested for observation and analysis. As shown in Figure 3a, the Sc0 cells mainly had nitrogen, carbon, and oxygen elements on their surfaces, with quite low levels of cadmium and sulfur elements. In contrast, the ScHB cells exhibited obvious signs of both sulfur and cadmium in the EDS images, indicating their enhanced heavy metal capture capabilities. Element quantification further confirmed the lower levels of carbon, together with the higher levels of cadmium and sulfur on the surface of the ScHB cells (Figure 3b,d). In detail, while the two strains had similar oxygen and nitrogen mass percentages (Figure S1a,b), the ScHB cells had 3.47% sulfur and 6.24% cadmium, while Sc0 only had 0.95% and 0.14% of these elements, respectively (Figure 3b,d). Taken together, these results confirmed the high cadmium-capturing capacity of the artificial ScHB cells.

### 3.3. The Artificial ScHB Cells Promote Plant Growth in Alum Mine Soils

**Figure 4:**
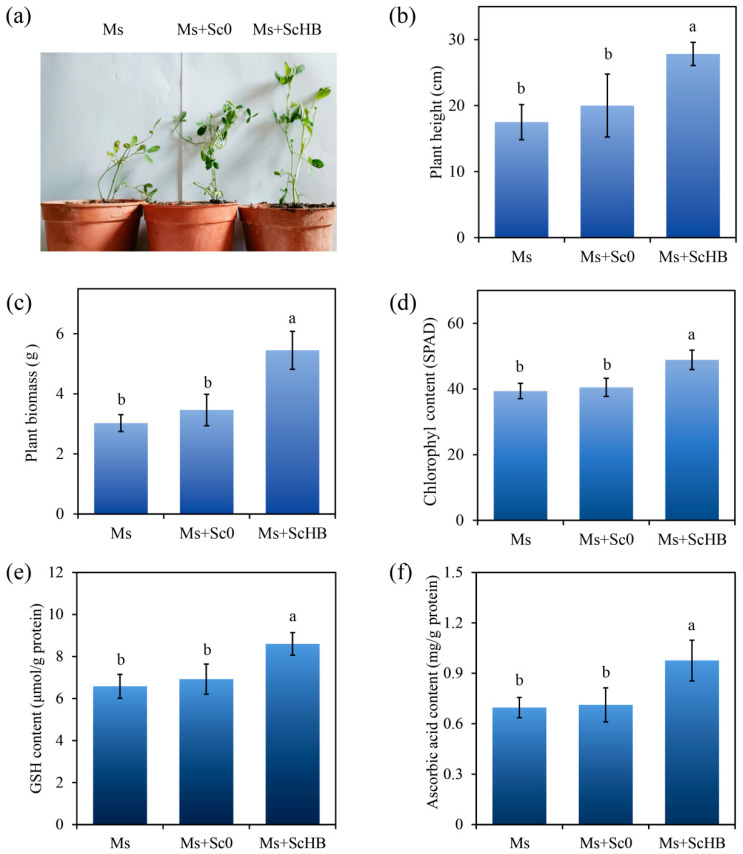
The growth of *M. sativa* plants cultured in alum mine soils. (**a**) Image of the plants. The young plants (height = 10 cm ± 1 cm) were cultured in the alum mine soils with no addition of the yeast cells (Ms), with the addition of Sc0 cells (1 × 10^8^ yeast cells/kg soil, Ms + Sc0), or the addition of Sc0 cells (1 × 10^8^ yeast cells/kg soil, Ms + Sc0). The plants were further cultured for 30 days and then photographed. (**b**) The height of the plants after 30 days. (**c**) The biomass of the plants. (**d**) The chlorophyl contents in the plant leaves. (**e**) The GSH contents in the leaves. (**f**) The ascorbic acid contents in the plant leaves. Different letters indicate significant differences between the groups (*p* < 0.05).

The effect of the yeast cells on plant growth in alum mine soils was further evaluated using *M. sativa* plants. After 30 days of culturing, the control plants (Ms) grew quite slowly, with the plant height only increasing from 10 cm to 17.5 cm (Figure 4a). The treatment with Sc0 did not significantly promote plant growth, with the plant height and biomass maintaining the same levels as those of the control group (Figure 4b,c). In contrast, the treatment with ScHB led to remarkable increases in both the plant height and biomass, with height increasing from 17.5 cm to 27.9 cm and biomass from 3.03 g to 4.35 g/plant (Figure 4b,c). Moreover, the ScHB group exhibited a higher leaf chlorophyll content than the Ms and Ms + Sc0 groups, with a SPAD value at 48.9 versus 39.4–40.5 (Figure 4d). Therefore, the addition of ScHB strongly promoted chlorophyll content and enhanced plant growth in the alum mine soils.

Glutathione (GSH) and ascorbic acid are two critical endogenous antioxidant agents for fighting against oxidative stress induced by osmotic pressure and heavy metals [35]. We then detected the levels of these two agents in the plant leaves. As shown in Figure 4e,f, the Ms + ScHB group had higher levels of these agents than the other two groups, with the GSH contents at 8.6 μmol/g protein versus 6.6–6.9 μmol/g protein and with the ascorbic acid contents at 0.98 mg/g protein versus 0.70–0.71 mg/g protein. These results indicate that the addition of ScHB enhanced the antioxidant capacity of the plants during phytoremediation.

### 3.4. The Artificial ScHB Cells Improve the Quality of Alum Mine Soils Cultured with M. sativa

**Figure 5:**
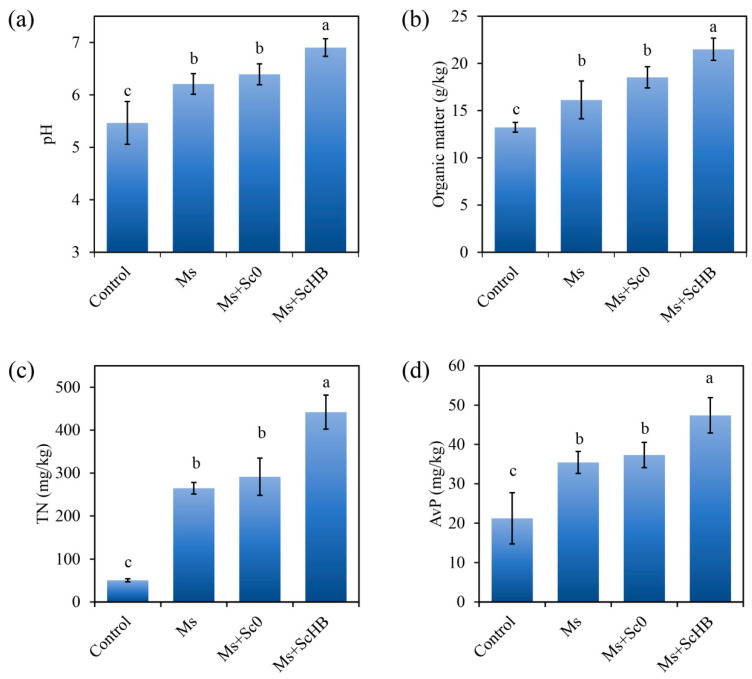
The quality of alum mine soils cultured with *M. sativa* (Ms, Ms + Sc0, Ms + ScHB) or not (control) after 30 days: (**a**) pH values. (**b**) Organic matter contents. (**c**) Total nitrogen (TN) contents. (**d**) Available phosphorus (AvP) contents. Different letters indicate significant differences between the groups (*p* < 0.05).

The soil quality is of significance for plant growth. To investigate whether the enhancement in plant growth is associated with soil quality, the chemical properties of the soils after 30 days of plant culturing were determined. While the control alum mine soils had an acidic pH value of 5.47, the soils after plant growth had improved pH values of >6.21. In particular, the soils from the Ms + ScHB treatment had the highest pH value, at 6.9 (Figure 5a), indicating that ScHB efficiently assisted the plant in neutralizing the soil acids. Furthermore, plant growth improved the soil organic matter, total nitrogen (TN), and available phosphorus (AvP) contents, with the Ms + ScHB treatment exhibiting the highest capacity to improve their values (Figure 5b,d). For example, while Ms and Ms + Sc0 only increased the soil organic matter contents from 13.2 g/kg to 16.1–18.5 g/kg, Ms + ScHB strongly increased the contents to 21.5 g/kg (Figure 5b). Similarly, Ms + ScHB led to an increase in TN contents from 50.7 mg/kg to 442.0 mg/kg and to an increase in AvP contents from 21.2 mg/kg to 47.4 mg/kg. In contrast, Ms and Ms + Sc0 only increased the TN and AvP contents to <291.4 mg/kg and <31.4 mg/kg, respectively (Figure 5c,d). Hence, the addition of ScHB drastically improved the soil quality of the alum mine soils during *M. sativa* culturing.

### 3.5. The Artificial ScHB Cells Increase Rhizosphere Bacterial Numbers in Alum Mine Soils

**Figure 6:**
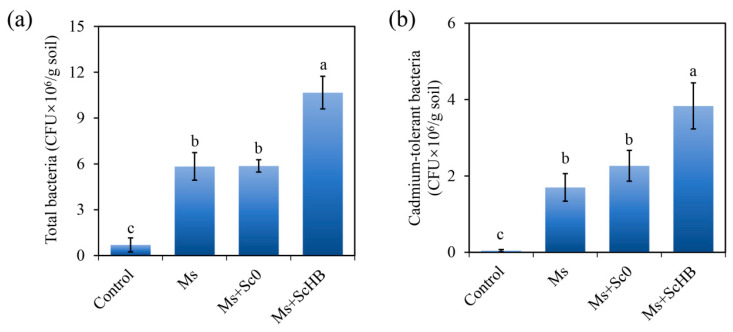
The number of total (**a**) and cadmium-tolerant (**b**) bacteria in the rhizosphere alum mine soils of *M. sativa*. The rhizosphere soils were sampled after 30 days of culturing to determine the bacterial numbers using CFU assays. Different letters indicate significant differences between the groups (*p* < 0.05).

Rhizosphere bacteria play a critical role in soil nutrient transformation and plant metabolism [36,37]. The number of total and cadmium-tolerant bacteria in the rhizosphere were then determined using colony-forming unit (CFU) assays. As shown in Figure 6, the plant treatments (i.e., Ms, Ms + Sc0, and Ms + ScHB) strongly increased the total bacterial numbers from 0.7 × 10^6^ CFU/g soil to >5.8 × 10^6^ CFU/g soil, and the cadmium-tolerant bacterial numbers from 0.1 × 10^6^ CFU/g soil to >1.7 × 10^6^ CFU/g soil. More strikingly, the Ms + ScHB group had the highest levels of both total and cadmium-tolerant bacteria, with the total bacterial numbers increasing to 10.7 × 10^6^ CFU/g soil (Figure 6a) and with the cadmium-tolerant bacterial numbers increasing to 3.8 × 10^6^ CFU/g soil (Figure 6b). These results revealed the positive effect of ScHB on the growth and colonization of bacteria in the plant rhizosphere soils.

### 3.6. The Artificial ScHB Cells Promote Removal of Heavy Metal Contents by the Plants

**Figure 7:**
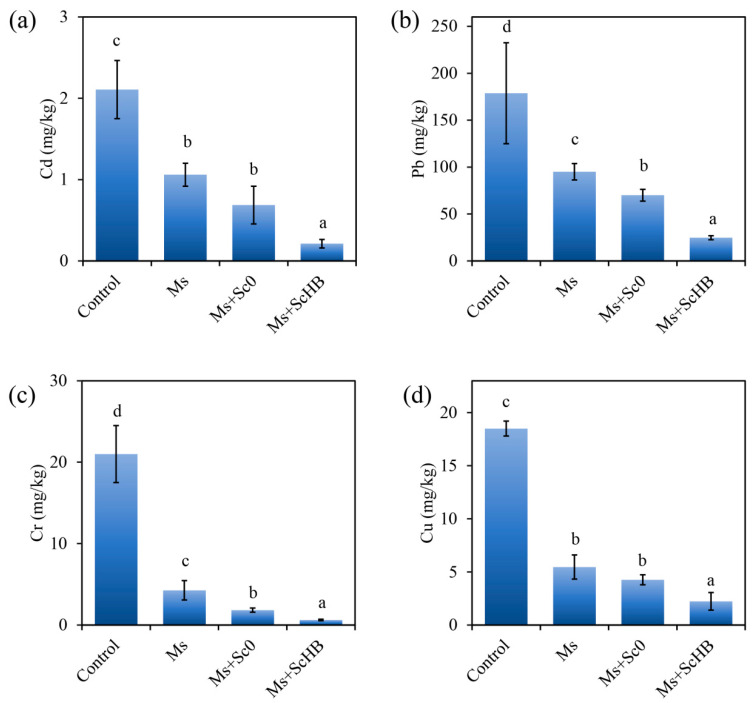
Heavy metal contents in the rhizosphere alum mine soils of *M. sativa* after 30 days of culturing. (**a**) Cadmium contents. (**b**) Lead contents. (**c**) Chromium contents. (**d**) Copper contents. Different letters indicate significant differences between the groups (*p* < 0.05).

Heavy metal removal is a representative phytoremediation process for alum mine soils [38]. The contents of heavy metals in rhizosphere soils were determined: while the untreated control soil maintained high levels of the tested heavy metals, i.e., cadmium, lead, chromium, and copper, the soils after plant culturing had significantly reduced contents of these heavy metals. In detail, the Ms and Ms + Sc0 treatment decreased the cadmium content from 2.1 mg/kg soil to 0.7–1.1 mg/kg soil, the lead content from 178.7 mg/kg soil to 70.0–95.0 mg/kg soil, the chromium content from 21.0 mg/kg soil to 1.8–4.3 mg/kg soil, and the copper content from 18.5 mg/kg soil to 4.3–5.5 mg/kg soil (Figure 7a–d). Moreover, the Ms + ScHB treatment removed the most soil heavy metals, achieving the lowest levels, with the soil cadmium, lead, chromium, and copper contents decreasing to 0.2 mg/kg soil (i.e., by 90%), 24.7 mg/kg soil (i.e., by 86%), 0.6 mg/kg soil (i.e., by 97%), and 2.2 mg/kg soil (i.e., by 88%) mg/kg soil, respectively (Figure 7a–d). This means that the Ms + ScHB treatment led to a remarkable decrease in heavy metal levels, which may be attributed to the accumulation of these heavy metals onto the yeast cells, followed by transport into the plant roots. Together, these results reveal that ScHB drastically enhanced the capacity of the plant to remove heavy metals from the alum mine soils.

## 4. Discussion

Alum mining has brought about great threats to the health of ecosystems and human beings. Abundant toxic agents, e.g., heavy metals, inorganic acids, and persistent organic matters, have been released from mine sites into peripheral soils and water, leading to serious environmental problems that need to be solved [39]. Phytoremediation is a green and economic strategy to treat mining pollution via plant transformation, hyperaccumulation, neutralization, etc. However, the efficiency of the used plants has frequently been compromised by their limited accumulation capacity and low tolerance to toxic agents. Herin, we developed an artificial yeast strain, which exposed the de novo designed heavy metal-capturing protein on the cell surface, to assist in the accumulation of heavy metals and promote plant tolerance to toxic agents. Therefore, the engineered yeast cells may be applied in the phytoremediation of alum mining soils to improve the soil quality in mining areas. Moreover, since the yeast cells could be easily engineered with no introduction of resistance markers, different artificial genes could be customized and transformed into the yeast cells for application in alum mines and other degraded areas containing multiple pollutants.

In this study, we found that the yeast cells exposing the heavy metal-capturing protein HBGFP exhibited a high capacity to capture heavy metals. In particular, during co-incubation of the engineered cells with the cadmium-containing medium, the cells had much higher levels of sulfur and cadmium than the control Sc0 cells (Figure 3). These results may be attributed to the strong exposure of the designed HBGFP, which is rich in sulfur-containing cystine. Commonly, cystine may bind strongly to free cadmium ion, endowing the yeast cells with the ability to adsorb cadmium. Furthermore, phytoremediation experiments revealed that the addition of the engineered yeast cells not only resulted in a remarkable decrease in cadmium levels but also reduced the levels of other heavy metals in the soils, including lead, chromium, and copper. This indicates that the yeast cells may also facilitate the transport of other heavy metals to the plants. This observation is consistent with our previous results showing that the engineered *Escherichia* cells exposing the heavy metal-capturing protein strongly capture a series of heavy metals [31].

After the addition of the engineered ScHB cells into the alum mining soils, the number of total and cadmium-tolerant bacteria increased remarkably in the rhizosphere soils of the *M. sativa* plants. This increase may be attributed to two possible mechanisms. One is that the yeast cells decreased the levels of heavy metals in the soils, leading to attenuated heavy metal stress around the plant roots and consequent growth of the bacterial cells. The other mechanism is that the yeast cells may recruit heavy metal-capturing bacteria that unite with the yeast cells to further attenuate the heavy metal levels, providing friendly sites for bacterial colonization and growth.

As revealed through the quantification of heavy metals in the rhizosphere soils, the ScHB cells strongly promoted heavy metal removal by *M. sativa*, leading to a remarkable decrease in heavy metal levels. Herein, we only determined the heavy metal levels after 30 days of culturing. Therefore, we cannot exclude the possibility that partially captured heavy metals may be released from the yeast cells and plant tissues after some more time passes. To further explore the sustainability of this bioremediation system, long-term monitoring of heavy metals should be performed in the future.

## 5. Conclusions

This study constructed the artificial yeast strain ScHB to enhance the phytoremediation of alum mine soils. The ScHB strain, which was transformed using a de novo synthesized gene containing surface-displaying sequences, a heavy metal-binding domain, and the fluorescence protein GFP, efficiently exposed the designed heavy metal-capturing protein HBGFP on its cell surface, leading to a high capacity to capture and tolerate cadmium. During the culturing of *M. sativa* in the alum mine soils, the addition of the ScHB cells strongly promoted plant growth and induced the accumulation of antioxidant agents in the plants. Furthermore, ScHB strongly improved the soil quality, with an increase in nutrient contents and soil bacterial counts. Furthermore, the ScHB cells and plants worked in combination to decrease the levels of cadmium, lead, chromium, and copper in the rhizosphere soils by 90%, 86%, 97% and 88%, respectively. This engineered yeast strain may improve the quality of alum mine soils through phytoremediation and may also be used in heavy metal enrichment during other kinds of bioremediation. In the future, the mechanism of yeast-cell-enhanced phytoremediation and the long-term effect of yeasts on alum mine soils will be further investigated.

## Data Availability

The original contributions presented in this study are included in the article/Supplementary Material. Further inquiries can be directed to the corresponding author.

## Supplementary Materials

The following supporting information can be downloaded at https://www.mdpi.com/article/10.3390/microorganisms13030612/s1, Figure S1: Percent of oxygen (O) and nitrogen (N) mass in the Sc0 and ScHB cells, revealed by EDS mapping analysis; Table S1: The sequence of the designed protein HBGFP.
