# Supplementary material for: An Engineered Yeast Expressing an Artificial Heavy Metal-Binding Protein Enhances the Phytoremediation of Alum Mine Soils
Source: Microorganisms. 2025 Mar 7;13(3):612. doi: 10.3390/microorganisms13030612 (PMC11944382; doi:10.3390/microorganisms13030612)
Supplement: Supplementary file 1 [file microorganisms-13-00612-s001.zip › microorganisms-3342272-supplementary.pdf]

microorganisms

MDPI

Supporting Information

Surface exposure of artificial heavy metal-binding protein on engineered yeast cells assists phytoremediation of alum mine soils

Wenming Wang, [1] Liling Xie, [2] Lin Zhao, [1] Qilin Yu[2,*]

[1] School of Environmental Science and Engineering, Tianjin University, Tianjin 300072, China
[2] National Key Laboratory of Intelligent Tracking and Forecasting for Infectious Diseases, College of Life Sciences, Nankai University, Tianjin, 300071, China
* Correspondence: yuqilin@mail.nankai.edu.cn (Y.Q.)

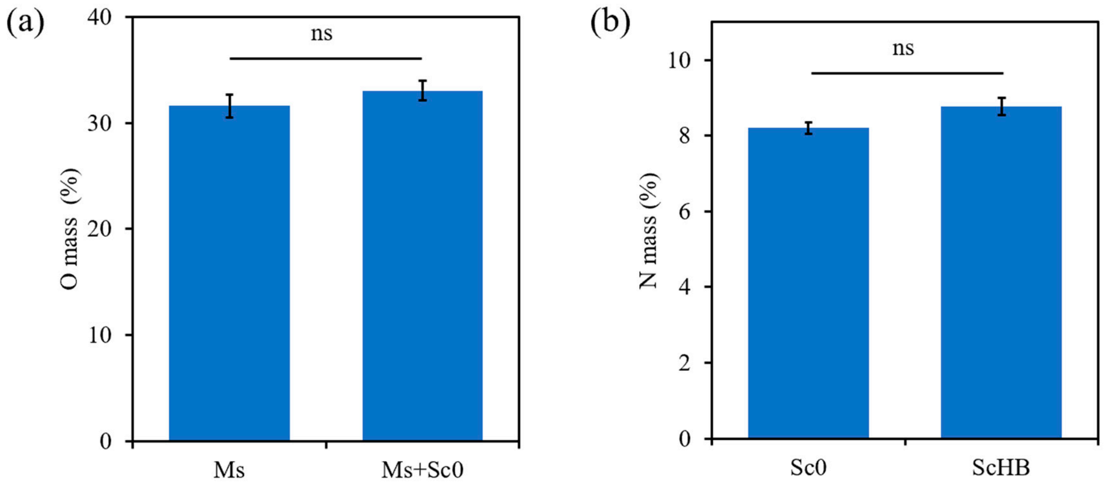

Figure S1. Percent of oxygen (O) mass and nitrogen (N) mass in the Sc0 and ScHB cells revealed by EDS mapping analysis.

Table S1. The sequence of the designed protein HBGFP

Name	Sequence*
HBGFP	1 MRFPSIFTAV LFAASSALAA PVNTTTEDET AQIPAEAVIG YLDLEGDFDV AVLPFSNSTN<br>61 NHHHHHHGGG CGCPCGCGGG CGCCGCGCCG CGCCGGGMDP NCSCTTGVSC ACTGSCKCKE<br>121 CKCTSCKKSC CSCCPVGCAK CAHGCVCKGT LENCSCCGGG VSKGEELFTG VVPILVELDG<br>181 DVNGHKFSVS GEGEGDATYG KLTLKFICTT GKLPVPWPTL VTTLTYGVQC FSRYPDHMKQ<br>241 HDFFKSAMPE GYVQERTIFF KDDGNYKTRA EVKFEGDTLV NRIELKGIDF KEDGNILGHK<br>301 LEYNYNSHNV YIMADKQKNG IKVNFKIRHN IEDGSVQLAD HYQQNTPIGD GPVLLPDNHY<br>361 LSTQSALSKD PNEKRDHMVL LEFVTAAGIT LGMDELYKIQ QNFTSTSLMI STYEGKASIF<br>421 FSAELGSIIF LLLSYLLF

*The sequences with purple, blue, green and red colors indicated the signal peptide of MFα1, the HB domain, the GFP sequence, and the GPI-targeting sequence of AGα1, respectively.
